# Effects of stand condition and root density on fine-root dynamics across root functional groups in a subtropical montane forest

**DOI:** 10.1007/s11676-022-01514-0

**Published:** 2022-07-23

**Authors:** Lin Huang, Rudong Zhao, Xiaoxiang Zhao, Qiuxiang Tian, Pengyun Yue, Feng Liu

**Affiliations:** 1 CAS Key Laboratory of Aquatic Botany and Watershed Ecology, Wuhan Botanical Garden, Chinese Academy of Sciences, Wuhan, 430074 People’s Republic of China; 2 University of the Chinese Academy of Sciences, Beijing, 100049 People’s Republic of China

**Keywords:** Root functional group, Root production, Root mortality, Root turnover, Stand condition, Badagongshan mountain

## Abstract

Fine roots play key roles in belowground C cycling in terrestrial ecosystems. Based on their distinct functions, fine roots are either absorptive fine roots (AFRs) or transport fine roots (TFRs). However, the function-based fine root dynamics of trees and their responses to forest stand properties remain unclear. Here, we studied the dynamics of AFRs and TFRs and their responses to stand conditions and root density in a subtropical montane mixed forest based on a 2-a root window experiment. Mean (± SE) annual production, mortality, and turnover rate of AFRs were 7.87 ± 0.17 m m^−2^ a^−1^, 8.13 ± 0.20 m m^−2^ a^−1^and 2.96 ± 0.24 a^−1^, respectively, compared with 7.09 ± 0.17 m m^−2^ a^−1^, 4.59 ± 0.17 m m^−2^ a^−1^, and 2.01 ± 0.22 a^−1^, respectively, for TFRs. The production and mortality of fine roots were significantly higher in high root-density sites than in low-root density sites, whereas the turnover of fine roots was faster in the low root-density sites. Furthermore, root density had a larger positive effect than other environmental factors on TFR production but had no obvious impact on AFR production. Tree species diversity had an apparent positive effect on AFR production and was the crucial driver of AFR production, probably due to a complementary effect, but had no evident impact on TFR. Both tree density and tree species diversity were positively correlated with the mortality of AFRs and negatively related to the turnover of TFRs, suggesting that higher root density caused stronger competition for rooting space and that plants tend to reduce maintenance costs by decreasing TFR turnover. These findings illustrated the importance of root functional groups in understanding root dynamics and their responses to changes in environmental conditions.

**Supplementary Information:**

The online version contains supplementary material available at 10.1007/s11676-022-01514-0.

## Introduction

Fine roots, defined as roots with a diameter ≤ 2 mm, provide essential functions including nutrient and water acquisition for plants (McCormack et al. 2017) and are a major contributor to ecosystem carbon sequestration through their rapid turnover and subsequent decomposition (Jackson et al. 1997). The amount of carbon cycled via fine-root production and decomposition is equal to or greater than that circulated through aboveground litters (Nadelhoffer and Raich 1992; Majdi and Pa 2005) and accounts for between 10% to over 50% of annual net primary productivity in terrestrial plant systems (Aerts et al. 1992; Ruess et al. 2003). Patterns of fine-root production, mortality, and turnover across root functional groups are considered important for plant ecology in mediating the carbon allocation strategy for the whole plant (McCormack et al. 2015).

Numerous studies have shown that root dynamics could be highly sensitive to environmental factors and plant community characteristics, such as soil nutrient contents (McNickle and Brown 2014; Majdi and Pa 2005; Kou et al. 2018), stand conditions (Brassard et al. 2011; Finér et al. 2011; Ma and Chen 2017; Lepik et al. 2020; Zeng et al. 2020), and competition with neighbors (Casper et al. 1997; Beyer et al. 2013; Cahill and James 2003). However, few studies have explored the divergent responses of fine roots between functional groups to environmental conditions and plant communities. These divergent responses reflect the differences in whole-plant strategies for resource allocation. They also represent a cost − benefit tradeoff between the construction of relatively inexpensive short-lived roots with a high capacity for acquisition and the production of long-lived roots with high costs for tissue defense and construction (Eissenstat and Yanai 1997; Kong et al. 2019). Therefore, understanding the divergence of fine-root dynamics across different fine-root functional types and the controlling factors in forest ecosystems can provide information on how the growth strategies of fine roots may respond to environmental changes.

Root density largely represents the remaining available space of roots per unit volume, which can be used as an indication of belowground competition among roots for space or resources. Since plants are sessile, fine roots are fundamentally constrained by the necessity to initiate all root growth from the fixed rooting point, most of their environmental interactions occur in a restricted space (Casper et al. 2003). Hence, the belowground space of root has been used to model and understand root system competition for soil nutrients, water, and foraging ability (Casper and Jackson 1997; Biondini 2001; Casper et al. 2003). Previous studies suggested that higher root density can result in more intense competition within the root system due to root foraging activity (Majdi and Persson 1993; Casper and Jackson 1997; Leuschner et al. 2001). In the field, roots may respond to the density of other roots, both interspecific and intraspecific (Herben et al. 2020). To preempt space and resources, vigorous root growth is often associated with strong competition (Baldwin 1976), especially under resource-limited soil conditions. Meanwhile, trees growing on nutrient-poor sites often give priority to invest a higher proportion of assimilated carbon (C) into roots to stimulate root growth (Bonifas et al. 2005; Prescott et al. 2020). Although many studies have focused on the mechanism underlying the response of fine-root production to root aggregation, few studies have addressed the response of fine-root dynamics to root density. The species composition of a plant community influences the nature and intensity of root interactions in the community, mediated by differences in rooting depth, root structures or root foraging behavior (Schenk 2006; Ma and Chen 2016). Studies have found that fine-root turnover rate increases with tree species richness in natural temperate forests (Jacob et al. 2014) and plantations (Lei et al. 2012). These studies attribute the high root turnover rate to intense competitive pressure in species mixtures (Jacob et al. 2014; Lei et al. 2012). However, such a pattern of higher root turnover rate in tree species mixture than in monoculture was not observed in a temperate plantation (Domisch et al. 2015). Despite this, the relationship between plant species diversity and root turnover is still elusive, in part because methodological limitations make root dynamics relatively difficult to estimate. Many studies have indicated that plant species diversity positively affects both above- and belowground productivity in multiple ecosystem types due to “overyielding” (Brassard et al. 2011; Ma and Chen 2016; Ouyang et al. 2019; Zeng et al. 2020). Two mechanisms have been proposed to explain this phenomenon. One hypothesis is the niche complementarity effect, which assumes that higher diversity could lead to greater productivity through niche differentiation and facilitation (Tilman et al. 1997; Marquard et al. 2009). Another hypothesis is the selection probability effect, which proposes that higher species richness improves productivity by increasing the chances of possessing high-yielding species (Špaèková and Lepš 2001; Schmid et al. 2008). Most research on the relationship between plant species diversity and root productivity has tended to support the complementarity effect rather than the selection probability effect (Brassard et al. 2011; Zeng et al. 2020; Jing et al. 2021).

Stand conditions such as tree density and species diversity in a forest ecosystem have strong influences on nutrient cycling and root dynamics. Tree density determines the spatial structure of a forest and can directly influence the distribution of light, heat, wet, and other environmental factors affecting aboveground productivity (Cai et al. 2016; Bo et al. 2018), which further indirectly affects belowground processes. Higher tree density can increase forest C storage and wood production because more light can be captured through higher canopy coverage (Morin 2015; Ouyang et al. 2019). The impact of tree density on root productivity has mostly been studied in relation to changes in species richness in natural forests (Marquard et al. 2009; Zeng et al. 2020). While the effects of species richness and tree density on root productivity are difficult to disentangle, higher species richness is usually accompanied by higher tree density (Marquard et al. 2009; Ouyang et al. 2019; Zeng et al. 2020). Recent research demonstrated that species richness was the most important factor in positively influencing fine-root production in a subtropical forest, tree density also had a marginal positive correlation to fine-root production, while the effect of species richness on fine-root production varied with tree density (Zeng et al. 2020). However, the effects of tree density and diversity on fine-root dynamics are still unclear in subtropical forests.

Fine roots of different functional types may respond differently to environmental conditions and stand characteristics due to their distinct adaptive strategies for resource acquisition and allocation. In many studies, fine roots were classified solely based on root diameter (< 2 mm), which may lead to high uncertainty in elucidating fine-root dynamics and their responses to environmental changes (Wang et al. 2019). In contrast, branch order is a fundamental architectural feature of roots that determines root morphological, chemical, and functional heterogeneity and is the strongest predictor of life span among all covariates (Wells and Eissenstat 2001; Pregitzer et al. 2002; Guo et al. 2008). Thus, a study based on fine-root functional types could more accurately estimate the root dynamics for a better understanding of their responses to environmental factors.

Fine roots can be separated into short-lived absorptive fine roots (AFRs) and long-lived transport fine roots (TFRs) based on their main functions (Xia et al. 2010; McCormack et al. 2015). AFRs (root orders 1–3) represent the most distal fine roots and are involved primarily in the acquisition and uptake of soil resources, whereas TFRs (root orders 4 and 5,) function primarily in structural support and transport with some storage capacity (Pregitzer et al. 2002; McCormack et al. 2015; Kou et al. 2018). In addition, roots of different functional types could have distinct responses to environmental change. For example, N deposition concurrently increases the production, mortality, and turnover of AFRs, but not TFRs (Kou et al. 2018). Meanwhile, AFRs are more responsive to environmental changes and turnover faster than TFRs (McCormack et al. 2015). However, we know little about how roots of different functional types may respond to environmental and stand conditions, especially in subtropical mixed forests. Here we investigated the dynamics of AFRs and TFRs in different stand conditions and root densities in a subtropical mixed mountain forest in China using root windows for more than two years. The objectives of this study were: (1) to estimate fine-root production, mortality, and turnover by root functional type; and, (2) to assess the production, mortality, and turnover of AFRs and TFRs at different root densities and stand conditions. Given the structural and functional differences between AFRs and TFRs, we hypothesized that (1) AFRs have greater production, larger mortality, and faster turnover than TFRs because AFRs are finer roots with greater absorptive abilities than TFRs. A fast turnover of AFRs helps plants to quickly acquire soil nutrients with relatively smaller expenses compared to TFRs. (2) The dynamics of AFRs and TFRs differ in response to stand condition and root density. Tree diversity is expected to have positive impacts on root production for both AFRs and TFRs due to complementary effects. Meanwhile, tree density and root density tend to have negative impacts on AFR production due to belowground competition, but probably not on TFR production because TFRs are relatively stable through time.

## Materials and methods

### Study site

The study was conducted in evergreen and deciduous broad-leaved mixed montane forest in the Badagongshan National Nature Reserve, Hunan Province (29°46.04' N, 110°5.24' E), in the mid-subtropical zone of China. The climate is subtropical mountain humid monsoon with an average annual precipitation of 2105.4 mm and annual relative humidity above 90%. The mean monthly air temperature ranges from 0.1 °C in January to 22.8 °C in July, with an annual mean of 11.5 °C. Soil pH ranges from 4.5 to 4.9. The monthly mean soil temperature ranged from 1.3 °C in January to 21.1 °C in July with an annual mean temperature of 10.7 °C. The dominant tree species at this study site include *Fagus lucida*, *Carpinus fargesii*, *Schima parviflora*, *Cyclobalanopsis multinervis*, and *Cyclobalanopsis gracilis* (Tian et al. 2017).

### Root window installation and image collection

We selected 10 forest plots (10 m × 10 m) dominated by *Fagus lucida*. The dynamics of fine roots were monitored using 40 cm × 40 cm root windows. In October 2017, two root windows were installed at 10–60 m apart and in different directions in every forest plot. Before root window installation, litter and about 2-cm layer of surface soil were carefully removed from the spot. The spot was then leveled with a shovel and covered with about 2 cm of sieved surface mineral soil (6 mm sieve) from the same plot. A 40 cm × 40 cm root window was then put roughly horizontal on each spot. All root windows were covered with black shade nets and then buried with a thin layer of soil and litter to prevent light and reduce temperature disturbance. Root scans began 6 months after root window installation to allow the soil around them to stabilize (Bai et al. 2010; Kou et al. 2018). Color root images were obtained monthly from April 2018 to August 2020 with a Canon 6D camera at a resolution of 2100 × 1575 pixels. Images were then cropped and adjusted to 400 dpi and calibrated in WinRhizo Tron (Regent Instruments Inc., Quebec City, Quebec, Canada) for further analysis. Images were not taken from December to March when snow usually blocked mountain roads or from December 2019 to July 2020 due to COVID-19. After the last sampling in August 2020, the root windows were removed. We marked all the roots in the windows and traced them to identifiable aboveground plant parts to determine the tree species, and the number of species was recorded. The roots within each window were collected intact and stored in bags in dry ice for later laboratory analysis.

### Field survey and soil sampling

All forest plots were surveyed in April 2018, and trees were measured. Trees with trunks higher than 3 m were counted to obtain the stand density per plot, and each tree was identified to species. Tree species richness was calculated using the Shannon − Wiener index to represent species diversity. Soil samples were extracted simultaneously near each root window for all plots. All soil samples were immediately sieved through a 2-mm mesh and stored at − 4 °C. Soil samples were tested for the presence of inorganic soil carbon, and there was no detectable inorganic carbon. Soil organic carbon and total nitrogen were measured with an elemental analyzer (Thermo Fisher Flash 2000, USA) interfaced with a Delta Plus Advantage mass spectrometer (Thermo Finigan, Bremen, Germany). Soil available phosphorus (P) was determined by NaHCO_3_ extraction with molybdenum anti-antimony colorimetry. The environmental characteristics of all plots are summarized in Table S1.

### Root image analysis and calculations

A total of 280 images were taken during the sampling and analyzed using WinRhizo Tron 2013c software (Regent Instruments) to measure root diameter and length and to distinguish root status (alive or dead), and color (white, brown, or black). We selected 13 root windows with the best root growth process presentation from all installed root windows for data analysis. We classified the white and brown-colored roots as living roots that have blackened and produced no new roots on subsequent occasions as dead roots. Dead roots were traced on all subsequent occasions until they became decayed and even disappeared completely from the images. The diameter and length of fine roots produced during the 1-month interval were estimated for each image, compared to the previous image and a new sequence of images. AFRs and TFRs can be easily distinguished by their distinct morphology; AFRs are smaller, generally dichotomously branched, and frequently form ectomycorrhizal structures, and the larger TFRs have a dark-red epidermis (McCormack et al. 2015). Roots were traced according to their order, new growths, old roots and dead roots. The branching number of fine roots was counted for the two functional groups as the number of branches per image area. The production rate (*R*_*p*_, m m^−2^ a^−1^), mortality rate (*R*_*m*_, m m^−2^ a^−1^), and turnover rate (*R*_*T*_, a^−1^) of fine roots were calculated as:

1 $${R}_{p}= {\mathrm{RL}}_{t+1}- {\mathrm{RL}}_{t} + {\mathrm{ARL}}_{t+1}$$

2 $${R}_{m}= {\mathrm{RDL}}_{t}- {\mathrm{RDL}}_{t+1}$$

RL_*t*_is the length of live roots at time *t*, RL_*t*+1_ is the length of previously imaged live roots at time *t* + 1, ARL_*t*+1_ is the length of new live roots at time *t* + 1. RDL_*t*_is the length of the roots with mortality at time *t*, RDL_*t*+*1*_ is the length of these roots at time *t* + 1. When RDL_*t*_ < RDL_*t*+1_, mortality is treated as 0 (Majdi and Pa 2005; Kou et al. 2018). Since root growth and mortality occur mainly at different times, root growth mostly occurs at the early and middle stages of the growing season while root death mostly occurs at the later stages.

3 $${R}_{T}=\frac{\mathrm{ARLP}}{\mathrm{ASRL}}$$

4 $$\mathrm{RLD}=\frac{{\mathrm{RL}}_{\mathrm{max}}}{A\cdot \mathrm{DOF}}$$

where ARLP is annual root length production per square meter, and ASRL is the average standing root length observed per square meter. RLD (m m^−3^) is the indicator of root density; RL_max_ is the maximum root length observed in the root window; *A* is the monitoring area (0.16 m^2^); DOF is the depth of focus with ranges from 5.61 to 5.92 m (Burton et al. 2000).

To examine the effects of root density on root dynamics, we used the maximum RLD occurring during the sampling period per year for each root window as the root density and separated it into two density levels (low as RLD < 3 m m^−3^ and high as RLD > 3 m m^−3^).

### Statistical analyses

For analyzing the dynamics of AFRs and TFRs in each root window, the annual fine-root production and mortality were calculated by multiplying the monthly average by 12. A one-way ANOVA was used to compare the differences between AFRs and TFRs. A two-way ANOVA was used to assess the differences in variables between AFRs and TFRs in the two root density conditions, then a post hoc least significant difference (LSD) test. Spearman’s rank correlation analysis was used to preliminarily examine the covarying environmental factors and the relation between all environmental variables and fine-root dynamics. We also used linear regression to examine the relationships between environmental factors and the dynamics of AFRs and TFRs. Variance partitioning analysis was used to quantify the relative importance of root density (RD), soil nutrients (SOC, TN, P), aboveground tree species richness, and tree density on fine root production around two root functional types. All the statistical analyses were implemented in R version 4.0.4 (R Core team, 2021).

## Results

### Production, mortality, and turnover of AFRs and TFRs

**Fig. 1:**
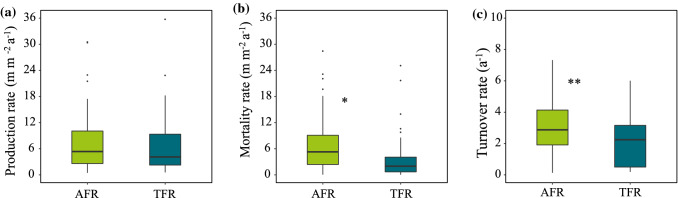
Comparisons of root (**a**) production rate, (**b**) mortality rate and (**c**) turnover rate between AFR and TFR. Boxes with asterisks indicate that the differences are significant, *P < 0.05, **P < 0.01. AFR: absorptive fine root, TFR: transport fine root

AFRs had higher mortality and faster turnover than TFRs (Fig. 1b, c; Table S2), but there was no significant difference in the production between AFRs and TFRs (Fig. 1a). Mean annual production, mortality, and turnover rate of AFRs was 7.87 ± 0.17 m m^−2^ a^−1^, 8.13 ± 0.20 m m^−2^ a^−1^, and 2.96 ± 0.24 a^−1^, respectively. Mean annual production, mortality, and turnover rate of TFRs was 7.09 ± 0.17 m m^−2^ a^−1^, 4.59 ± 0.17 m m^−2^ a^−1^, and 2.01 ± 0.22 a^−1^, respectively.

### Dynamics of AFRs and TFRs at different root densities

**Fig. 2:**
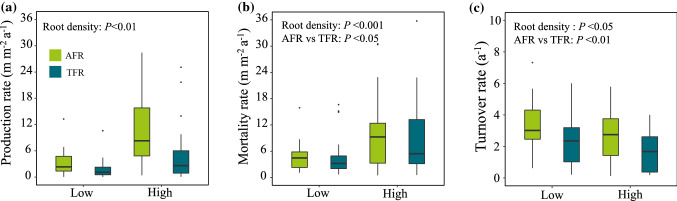
Comparisons of root (**a**) production rate, (**b**) mortality rate and (**c**) turnover rate between AFR and TFR for the low and high root density sites. Significant differences at *P* < 0.05. AFR: absorptive fine root, TFR: transport fine root

At the low root density level, the annual production, mortality, and turnover of fine roots ranged from 3.88 to 6.25 m m^−2^ a^−1^, 1.38 to 3.88 m m^−2^ a^−1^, 1.94 to 3.71 a^−1^, respectively, while the production, mortality, and turnover of fine roots at high root density ranged from 7.5 to 12.5 m m^−2^ a^−1^, 4.56 to 14 m m^−2^ a^−1^, 1.51 to 3.01 a^−1^, respectively (Table S3). The fine-root mortality was significantly greater at high root density sites than at low root-density sites, but the fastest turnover was in the low root-density sites (Fig. 2b, c).

There was no significant distinction in fine-root production between the two root functional types under both root density conditions (Fig. 2a), whereas the mortality of AFRs was dramatically higher than TFRs only at the high root density sites (Fig. 2b). In addition, the turnover of AFRs was faster than that of TFRs (Table S3).

### Relative contributions of factors on fine-root production

**Fig. 3:**
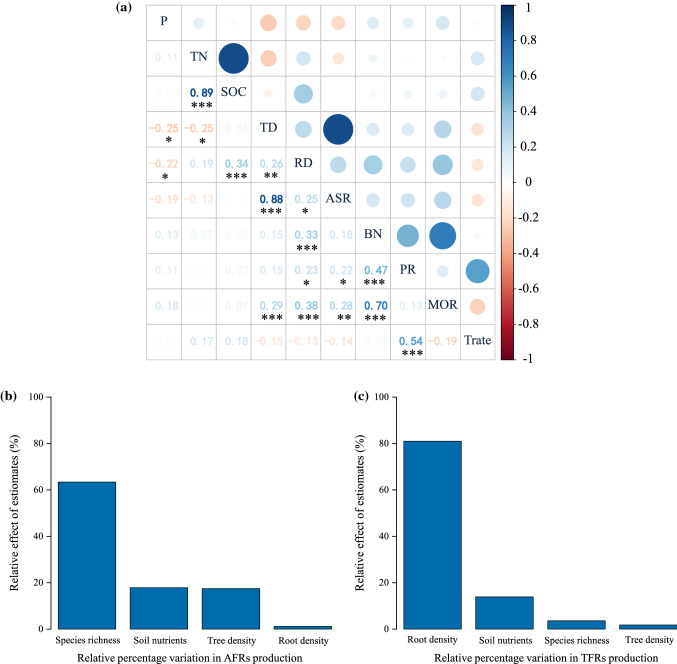
Pairwise correlations among all variables across both functional types (**a**). Asterisks indicate that the correlations are significant, *P < .05, **P < .01, ***P < .001. The digit of the square is proportional to the correlation coefficient. Relative percentage variation in the root production of AFRs (**b**) and TFRs (**c**) explained by root density, aboveground density, aboveground species richness and soil nutrients in the variance partitioning analysis. P: phosphorus, TN: total nitrogen, SOC: soil organic carbon, TD: tree density, RD: root density, ASR: aboveground species richness, BN: branching number, PR: root production, MOR: root mortality, Trate; root turnover rate

For both functional types of fine roots, root density had the highest significant effect on fine-root production, branching number, and mortality (Fig. 3a). Annual fine-root production and mortality were also positively associated with branch number. Fine-root turnover rate was positively correlated with fine-root production. Tree species richness and tree density, total nitrogen, and soil organic carbon were highly correlated variable pairs, respectively.

Tree species richness was the crucial driving factor of AFR production in the variance partitioning analysis (Fig. 3b). All predictors explained up to 38% (adjusted *R*^2^) of the total variation observed in AFR production, ranked in decreasing order of relative importance as tree species richness (63.4%, *R*^2^ = 0.28), soil nutrients (17.9%, *R*^2^ = 0.12), tree density (17.5%, *R*^2^ = 0.07) and root density (1.2%, *R*^2^ = 0.01). All the variables explained up to 18% of the variation in TFRs production (Fig. 3c), ranked in decreasing order of relative importance, were root density (81%, *R*^2^ = 0.11), soil nutrients (13.9%, *R*^2^ = 0.05), Tree species richness (3.6%, *R*^2^ = 0.01) and aboveground density (1.8%, *R*^2^ = 0.01). Root density had larger effects than other environmental factors on TFRs production.

### Correlations between environmental factors and the dynamics of AFRs and TFRs

**Fig. 4:**
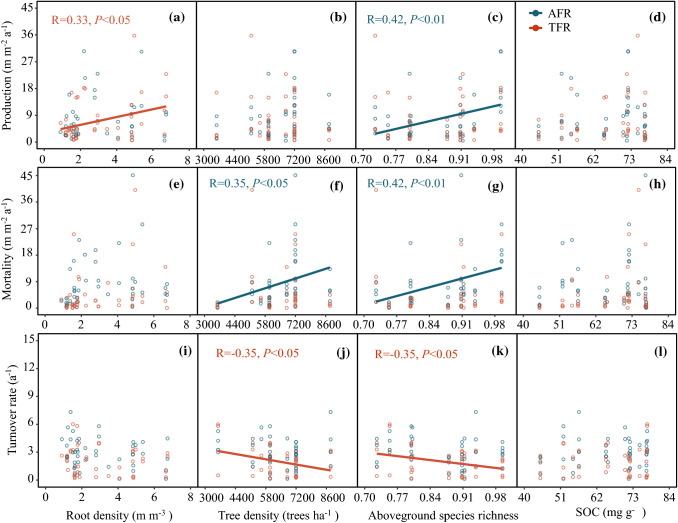
The relationships between root density, aboveground density, aboveground species richness and SOC with fine root production (**a**–**d**), mortality (**e**–**h**) and turnover rate (**i**–**l**) for both AFRs (blue circles) and TFRs (red circles), solid lines depict linear regressions. AFR: absorptive fine root, TFR: transport fine root

Tree species richness had significant positive effects on AFR production (Fig. 4c). In addition, root density positively affected TFRs production (Fig. 4a). Both tree density and species richness had apparent positive effects on the mortality of AFRs (Fig. 4f, g). However, tree density and species richness negatively affected the turnover of TFRs (Fig. 4j, k). SOC had no significant effect on fine-root dynamics (Fig. 4d, h, l).

## Discussion

### Production, mortality, and turnover of AFRs and TFRs

AFRs had a significantly greater mortality and turnover rate than TFRs. Many previous studies have reported that AFRs have greater mortality and turnover than TFRs (Goebel et al. 2011; McCormack et al. 2015; Kuo et al. 2018; Wang et al. 2019). AFRs have more root branches and larger absorptive area compared to TFRs, providing greater potential for uptake and mycorrhizal colonization. AFRs often form ephemeral root nodules characterized by a relatively short life span (Xia et al. 2010; McCormack et al. 2015). The AFRs are the most dynamic and responsive portion of the fine-root system (Guo et al. 2004; Kuo et al. 2018). Therefore, the greater mortality and faster turnover of AFRs can accelerate exogenous carbon input into the soil carbon pool, promoting the soil carbon cycle (Guo et al. 2004, 2008; Goebel et al. 2011). These results support our first hypothesis that AFRs and TFRs differ in mortality and turnover rate. However, there is no significant difference in root production between AFRs and TFRs.

### Effects of root density on the dynamics of AFRs and TFRs

**Fig. 5:**
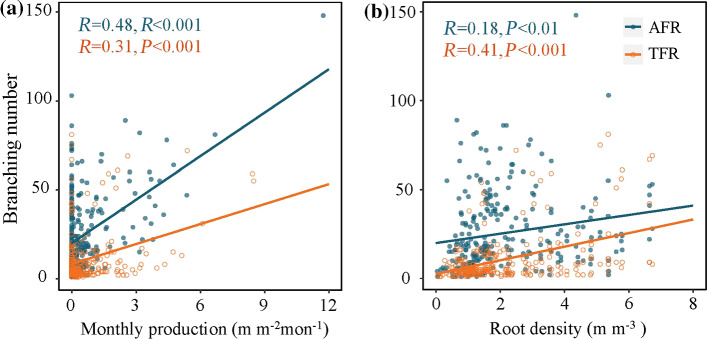
The relationships between monthly fine root branching number and monthly root production (**a**) and belowground root density (**b**) for both AFRs (blue circles) and TFRs (red circles), solid lines depict linear regressions. AFR: absorptive fine root, TFR: transport fine root

Fine-root production and mortality were greater at sites with high root density, whereas the faster fine-root turnover occurred at the low root density sites (Fig. 2). The covariation in the AFRs and TFRs at the two densities showed a consistent trend. The number of branches for both AFRs and TFRs increased dramatically as fine-root production and root density increased (Fig. 5). Plants usually allocate more C to the growth of fine roots to occupy belowground space and to enhance resource acquisition under belowground competition as root density increases. Accordingly, fine-root mortality may depend more on the magnitude of roots produced, since mortality is generally a passive process (Kou et al. 2018). The increase in fine-root mortality under high root density may be due to the greater fine-root production and stronger competition.

We further analyzed the predictors of AFRs and TFRs to determine whether root density plays a key role in triggering their dynamics. Root density had no distinct impact on the mortality and turnover of either the AFRs or the TFRs (Fig. 4e, i), inconsistent with our second hypothesis. On the other hand, as we hypothesized, higher root density had a significant and positive effect on TFR production, but no obvious impact on AFR production (Fig. 4a). This response to increased competitive pressure in the rooting zone may reveal a strategy of roots withdrawing from the periphery of the foraging area and increasing intra-root branching and production in the remaining area (Lepik et al. 2020). This discrepancy in the effects on AFRs and TFRs might be explained by the preemption of growth space tending to stimulate TFR production but has no decisive effect on AFR production. After all, TFRs are relatively stable through time and serve primarily as a physical structure with some storage capacity (McCormack et al. 2015). This function of TFR also suggests that higher root density probably leads to competition for growth space rather than increased resource acquisition. This conclusion can be partially explained by the fact that soil nutrients are positively related to root density but have no prominent impact on fine-root dynamics within a certain range (Figs. 3a, 4d, h, i).

### Impacts of stand conditions on the dynamics of AFRs and TFRs.

Tree species richness had obvious positive effects on root production and mortality of AFRs, but no evident impact on TFRs (Fig. 4c, g), supporting our second hypothesis. Many studies have demonstrated belowground “over-yielding” in species mixtures compared to monocultures (Brassard et al. 2013; Jacob et al. 2014; Ravenek et al. 2014; Ma and Chen 2018; Zeng et al. 2020), indicating that soil space is more fully occupied by fine roots in tree mixtures than in single-species monocultures (Brassard et al. 2011). Over-yielding reflects substantial rooting plasticity in response to neighbors (Schmid and Kazda 2005; Bolte and Villanueva 2006). Several studies have confirmed that variation in root traits as the diversity of neighbors increases leads to niche differentiation and complementary colonization of underground space, thus promoting soil resource acquisition and reducing the competitive pressure among neighbors (Brassard et al. 2013; Mueller et al. 2013; Ma and Chen 2017; Jing et al. 2021). Studies also have revealed evidence of interactive effects between nutrients and neighbors where some plants increase their root production in the presence of competition from neighbors (Mommer et al. 2010; Padilla et al. 2013). Our results are partially consistent with these studies that root systems preferentially allocate more C to the construction of AFRs with increasing tree species diversity, thus improving foraging efficiency and eventually increasing total resource uptake. Thus, the increase in AFRs mortality with increasing species richness could have been simply due to the higher production of AFRs (Kou et al. 2018).

Tree species richness differentially influenced the turnover of TFRs (negative effect) and AFRs (no effect), opposite the faster fine-root turnover found as species richness increased in temperate forests (Lei et al. 2012; Jacob et al. 2014; Ma and Chen 2018). The amount and timing of root production and mortality codetermine fine-root turnover rate by affecting annual cumulative production and the mean standing length (Brunner et al. 2013). We noticed a significant positive correlation between fine-root production and turnover rate (Fig. 3a). Tree species diversity promoted the production and mortality of AFRs, which partly explained why tree species diversity had no apparent influence on the turnover of AFRs.

We found that tree density was positively correlated with the mortality of AFRs and negatively related to the turnover of TFRs, but not related to root production (Fig. 4b, f, j). The nonsignificant relationship between tree density and root production differs from recent reports showing that fine-root biomass is lower in a recently thinned stand compared to a dense old coppice (Montagnoli et al. 2012a, b), and another study also found a slight positive correlation between tree density and fine-root production (Zeng et al. 2020). In mixed forests, both tree C storage and aboveground net primary productivity were significantly influenced by the combination of stand density and species richness (Cai et al. 2016). The difference in root mortality and turnover in response to tree density between AFRs and TFRs may be due to aboveground competition caused by the combination of higher density of stems and abundant tree species diversity, which further affects root allocation strategy. Therefore, to interpret fine-root dynamics, tree density needs to be assessed in conjunction with changes in tree species richness.

## Conclusion

Fine-root production did not differ significantly between AFRs and TFRs, nor did root density significantly affect the production of either type. Mortality of AFRs was significantly greater than the TFRs, especially in the high root density sites. The turnover of AFRs was faster than TFRs, especially in low root density sites. The production and mortality of fine roots were higher in high root density sites, whereas fine-root turnover was faster in the low root density sites. Furthermore, root density had a larger positive effect on TFRs production than other environmental factors, but had no obvious impact on AFRs production. Our findings confirmed that the denser root density leads to competition for rooting space rather than increased resources acquisition and stimulates plants to allocate more C to the proliferation of stable TFRs. Tree species diversity had an apparent positive effect on AFRs production and was the crucial driver of AFRs production, but no evident impact on TFRs, probably due to niche complementation. With increasing tree species diversity, the root system prioritizes the construction of cheaper AFRs of fast foraging and short lifespan. Moreover, both tree density and species diversity were positively correlated with AFRs mortality, and negatively related to TFRs turnover. Higher root mortality of AFRs under higher tree density and species diversity was consistent with the higher production of AFRs. These findings indicate that a more mechanistic understanding of fine-root dynamics and its response to environmental conditions require more root-order-based functional experiments. Studies based on root hierarchical systems and root functional types could improve our understanding of plant strategies for resource allocation and root dynamics.

## Supplementary Information

Below is the link to the electronic supplementary material.

Supplementary file1 (DOCX 24 KB)
