# Supplementary material for: Effects of stand condition and root density on fine-root dynamics across root functional groups in a subtropical montane forest
Source: J For Res (Harbin). 2022 Jul 23;34(3):665–75. doi: 10.1007/s11676-022-01514-0 (PMC9307969; doi:10.1007/s11676-022-01514-0)
Supplement: Supplementary file 1 — Supplementary file1 (DOCX 24 KB) [file 11676_2022_1514_MOESM1_ESM.docx]

**Table S1** Environmental characteristics for all the plots

| Root  density  levels | Win  -number | Win-root  density  (m m^−3^) | Soil-N  (mg g^−1^) | Soil-C  (mg g^−1^) | Soil-P  (mg kg^−1^) | Tree  species  richness | Shrub  species  richness | Tree  density  (ind. ha^−1^) | Basal  area  (m^2^ ha^−1^) | Win  -species |
| --- | --- | --- | --- | --- | --- | --- | --- | --- | --- | --- |
|  |  |  |  |  |  |  |  |  |  |  |
| Low  density | 1 | 0.796 | 6.2 | 70.6 | 5.55 | 0.8 | 0.68 | 5600 | 10.59 | 1 |
|  | 2 | 1.839 | 5.1 | 65.4 | 6.27 | 0.73 | 0.49 | 5200 | 7.24 | 1 |
|  | 3 | 1.273 | 5.6 | 77.3 | 4.51 | 0.75 | 0.47 | 3600 | 12.43 | 2 |
|  | 4 | 1.878 | 5 | 64.8 | 5.13 | 0.8 | 0.25 | 6000 | 12.41 | 3 |
|  | 5 | 1.957 | 3.7 | 44.9 | 5.13 | 0.88 | 0.67 | 6000 | 26.79 | 2 |
|  | 6 | 2.103 | 4.3 | 56.6 | 4 | 0.94 | 0.71 | 8800 | 14.57 | 2 |
| High  density | 7 | 3.199 | 5.1 | 72.1 | 5.34 | 0.99 | 0.79 | 7200 | 12.6 | 2 |
|  | 8 | 3.461 | 4.5 | 54.8 | 5.03 | 0.91 | 0.67 | 7200 | 12.14 | 2 |
|  | 9 | 4.257 | 3.6 | 51.9 | 4.51 | 0.8 | 0.25 | 6000 | 12.41 | 4 |
|  | 10 | 4.37 | 5.5 | 77.1 | 4.72 | 0.91 | 0.67 | 7200 | 12.14 | 4 |
|  | 11 | 4.504 | 5.5 | 75 | 5.86 | 0.73 | 0.49 | 5200 | 7.24 | 2 |
|  | 12 | 4.842 | 5.1 | 72.1 | 5.34 | 0.99 | 0.79 | 7200 | 12.6 | 3 |
|  | 13 | 5.599 | 5.3 | 71.7 | 3.79 | 0.91 | 0.53 | 6800 | 14.53 | 2 |

**Table S2.** Mean production, mortality and turnover rate of AFRs and TFRs.

| Root  type | Production  (m m^−2^ a^−1^) | | Morality  (m m^−2^ a^−1^) | | Turnover rate  (a^−1^) | |
| --- | --- | --- | --- | --- | --- | --- |
|  | Mean | SE | Mean | SE | Mean | SE |
| AFRs | 7.87a | 0.17 | 8.13a | 0.20 | 2.96a | 0.24 |
| TFRs | 7.09a | 0.17 | 4.59b | 0.17 | 2.01b | 0.22 |

The different letters indicate significant differences at *p*< 0.05

**Table S3.** AFRs vs TFRs root production (R^2^=0.095), branch number (*R*^2^=0.095), mortality (*R*^2^=0.187) and turnover rate (*R*^2^=0.083) at the low and high belowground root density conditions.

| Density level | Root  type | Production  (m m^−2^ a^−1^) | | Mortality  (m m^−2^ a^−1^) | | Turnover rate  (a^−1^) | |
| --- | --- | --- | --- | --- | --- | --- | --- |
|  |  | Mean | SE | Mean | SE | Mean | SE |
| Low density | AFR | 4.73a | 0.18 | 3.37a | 0.15 | 3.36a | 0.35 |
|  | TFR | 4.92a | 0.25 | 1.89a | 0.12 | 2.27b | 0.33 |
| High density | AFR | 10.49a | 0.36 | 12.10a | 0.42 | 2.69a | 0.32 |
|  | TFR | 8.90a | 0.36 | 6.82b | 0.40 | 1.79a | 0.28 |

The different letters indicate significant differences at *p* < 0.05.
